# Extracellular vesicle biomarkers: current status and future perspectives as novel tools in liquid biopsy

**DOI:** 10.3389/fimmu.2026.1853671

**Published:** 2026-05-19

**Authors:** Wenlong Huang, Minghong Zhao, Ling Li, Qun Chen, Mao Yang, Chun Zhou, Maoji Qi, Yanxia Wang, Liya Luo, Wanneng Liang, Zonghong Lai, Shenglan Pu, Defa Huang

**Affiliations:** 1 Department of General Medicine, The First People’s Hospital of Zunyi (Third Affiliated Hospital of Zunyi Medical University), Zunyi, China; 2 Laboratory Medicine, Guizhou Aerospace Hospital, Zunyi, China; 3 Burn Department, The First Affiliated Hospital of Gannan Medical University, Ganzhou, China; 4 Laboratory Medicine, The First Affiliated Hospital of Gannan Medical University, Ganzhou, China

**Keywords:** biomarkers, clinical translation, extracellular vesicles, isolation techniques, liquid biopsy, multi-omics

## Abstract

Extracellular vesicles (EVs) are nano-sized vesicles secreted by living cells and ubiquitously present in body fluids. They carry molecular cargo-including proteins, nucleic acids, and lipids-that accurately mirrors the physiological and pathological state of their parent cells, offering a highly promising novel source of biomarkers for liquid biopsy. This review systematically summarizes the progress in biomarker discovery and application of EVs in major diseases such as cancer, neurological disorders, and cardiovascular diseases. It provides an in-depth analysis of the key technical bottlenecks in current EV isolation, characterization, and detection methodologies, and discusses the challenges related to standardization and clinical translation. Finally, the review outlines the broad future prospects in this field, including integrated multi-omics analysis based on EVs, and their potential for early diagnosis, real-time therapeutic monitoring, and prognostic assessment.

## 1 Introduction

Extracellular vesicles (EVs) are secreted by virtually all living cells and are ubiquitously present in every human body fluid, serving as critical mediators of intercellular communication (1). These heterogeneous particles, varying in biogenesis, size, and composition, encapsulate a diverse molecular cargo-including proteins, nucleic acids (DNA and RNA), lipids, and metabolites-that faithfully reflects the molecular signature of their parental cells (2, 3). This inherent property positions EVs as powerful “molecular snapshots” or “treasure chests” of cellular state, offering an unprecedented window into physiological and pathological processes (4). In the context of cancer, numerous studies have demonstrated that EV-associated biomolecules can represent malignant phenotypes, making them increasingly discussed as valuable carriers of cancer biomarkers in liquid biopsy samples (1). The lipid bilayer membrane of EVs not only protects their cargo from degradation in the extracellular milieu but also facilitates their stable circulation in biofluids, enhancing their utility as reliable biomarker sources compared to more labile analytes (3, 5).

The field of liquid biopsy, which aims to non-invasively diagnose and monitor diseases through the analysis of biofluids, has traditionally focused on circulating tumor cells (CTCs) and circulating tumor DNA (ctDNA) (6, 7). However, EVs possess unique advantages that have propelled them into the spotlight as a “main third component” of liquid biopsy (4). Unlike CTCs, which are rare, or ctDNA, which can be fragmented, EVs are abundant, widely distributed, and their lipid membrane confers stability to their contents (3, 5). Furthermore, EVs carry a rich and complex molecular payload that includes not only DNA but also various RNA species (e.g., mRNA, microRNA, long non-coding RNA, circular RNA), proteins, and lipids, providing a multi-analyte platform for biomarker discovery (3, 8). **Table 1** compares various indicators of EVs, ctDNA and CTCs as liquid biopsy methods. This multi-faceted cargo allows for a more comprehensive molecular profiling of tumors, capturing information on gene expression, mutations, and post-translational modifications that may be missed by analyzing a single analyte type (9). For instance, in prostate cancer, EV biomarkers in blood and urine samples offer a novel and less-invasive alternative to surgical biopsies, potentially enabling clinicians to obtain a holistic picture of the tumor through a simple liquid sample (10). Beyond their value as passive biomarker carriers, EVs are increasingly recognized as active regulators of cancer immunity. Tumor-derived EVs can remodel the tumor immune microenvironment by transferring immunosuppressive proteins, immune checkpoint molecules, nucleic acids, metabolites, and bioactive lipids to immune cells. For example, EV-associated PD-L1, TGF-β, FasL, NKG2D ligands, and immunomodulatory microRNAs have been implicated in T-cell exhaustion, natural killer cell dysfunction, macrophage polarization, dendritic cell impairment, and expansion of myeloid-derived suppressor cells. Conversely, immune-cell-derived EVs may participate in antigen presentation, immune activation, or immune suppression depending on their cellular origin and molecular cargo. Therefore, EVs represent not only a source of cancer biomarkers but also a mechanistic interface between tumor evolution and host immunity. This immunological dimension is particularly relevant in the era of immune checkpoint blockade, adoptive cell therapy, and cancer vaccines, where EV-associated molecules may provide dynamic readouts of immune escape, treatment response, and resistance.

**Table 1:** Comparison of extracellular vesicles, ctDNA and CTCs as liquid biopsy analytes.

Feature	Extracellular vesicles (EVs)	Circulating tumor DNA (ctDNA)	Circulating tumor cells (CTCs)
Analyte type	Membrane-enclosed vesicles released by tumor and non-tumor cells; carry proteins, lipids, DNA, mRNA, miRNA, lncRNA, circRNA, metabolites, and surface markers	Fragmented tumor-derived DNA released into circulation mainly through apoptosis, necrosis, or active secretion	Intact viable or apoptotic tumor cells shed from primary or metastatic lesions into blood
Biological information captured	Multi-omic information, including genomic, transcriptomic, proteomic, lipidomic, metabolic, and immune-regulatory signals; reflects tumor cells and tumor microenvironment	Mainly genomic information, including mutations, copy number alterations, methylation, fragmentomics, and tumor burden	Cellular phenotype, morphology, protein expression, RNA profiles, genomic alterations, viability, epithelial–mesenchymal transition, and metastatic potential
Stability in biofluids	Relatively high stability because the lipid bilayer protects cargo from enzymatic degradation; detectable in plasma, serum, urine, CSF, saliva, bile, ascites, and other biofluids	Moderate stability; short half-life enables real-time monitoring but low abundance and pre-analytical degradation can be challenging	Low abundance and fragile; viability and integrity are affected by collection tubes, processing time, shear stress, and enrichment methods
Early detection sensitivity	Potentially high, especially in anatomically proximal biofluids such as urine for bladder/prostate cancer, CSF for brain tumors, bile or pancreatic juice for pancreatobiliary cancers; tumor-derived EVs may be released actively even from small lesions	Variable and often limited in early-stage or low-shedding tumors because ctDNA fraction can be extremely low; sensitivity improves with methylation or multi-analyte approaches	Generally limited for early detection because CTCs are rare, especially in localized disease; more informative in advanced or metastatic cancer
Specificity considerations	Cargo may be influenced by inflammation, tissue injury, infection, benign diseases, platelet activation, and normal-cell-derived EVs; tumor-specific EV enrichment remains challenging	High specificity for tumor mutations when known driver alterations are detected, but clonal hematopoiesis can cause false positives; methylation signatures require careful validation	High tumor specificity when CTC identity is confirmed, but enrichment bias and epithelial marker loss during EMT may reduce detection
Technical complexity	Moderate to high; requires standardized EV isolation/enrichment, particle characterization, cargo profiling, and normalization; methods include ultracentrifugation, size-exclusion chromatography, precipitation, microfluidics, and immunoaffinity capture	Moderate; established workflows include digital PCR, targeted NGS, whole-genome sequencing, methylation sequencing, and fragmentomics; analytical sensitivity is critical	High; requires rare-cell enrichment, identification, single-cell analysis, and preservation of cell integrity; epithelial-marker-based capture may miss mesenchymal CTCs
Cost and scalability	Currently variable; simple isolation methods are inexpensive but less specific, whereas immunocapture, microfluidics, single-EV analysis, and multi-omics profiling increase cost; scalability is improving but not yet uniform	Increasingly scalable; targeted assays are relatively cost-effective, whereas broad NGS and methylation assays are more expensive; several commercial platforms are mature	Typically expensive and labor-intensive due to rare-cell isolation, imaging, enumeration, and single-cell molecular assays; lower throughput than ctDNA
Clinical applications	Emerging applications in early detection, molecular subtyping, immune profiling, treatment response monitoring, minimal residual disease, recurrence warning, and biofluid-specific diagnostics	Established or rapidly expanding applications in mutation detection, therapy selection, resistance monitoring, MRD detection, recurrence surveillance, and tumor burden assessment	Established mainly for prognostic enumeration in selected cancers; molecular characterization and drug testing are promising but less widely adopted
Clinical/regulatory status	Mostly investigational; few EV-based assays have reached broad clinical implementation; standardization and prospective validation remain major barriers	Most clinically mature among the three; several FDA-approved or guideline-supported plasma ctDNA assays exist for therapy selection and monitoring in multiple cancers	Some regulatory-cleared platforms exist, such as CellSearch for CTC enumeration in metastatic breast, prostate, and colorectal cancers, but broader molecular applications remain investigational
Unique advantages	Captures protected multi-omic cargo and surface phenotype; reflects both tumor biology and tumor microenvironment; suitable for diverse biofluids and repeated sampling; may improve early detection through proximal fluid sampling; can provide immune-related information such as EV PD-L1 and immunomodulatory cargo	High analytical specificity for genomic alterations; well suited for detecting actionable mutations, resistance mutations, and MRD when tumor DNA is shed into circulation	Provides intact-cell information, allowing assessment of morphology, viability, heterogeneity, EMT status, and functional analyses such as ex vivo culture or drug sensitivity testing
Main limitations	Lack of standardized isolation and analysis; difficulty distinguishing tumor-derived EVs from abundant normal EVs and contaminants; limited large-scale prospective validation	Low sensitivity in low-shedding or early-stage tumors; clonal hematopoiesis confounding; limited protein/RNA/immune phenotype information	Extremely rare events, enrichment bias, high cost, low reproducibility across platforms, and limited sensitivity in early disease

In this review, this review focuses on EV-associated biomarkers at the interface of liquid biopsy, cancer immunity, and immunotherapy. Unlike previous EV-liquid biopsy reviews that primarily summarized isolation methods, general cargo, or diagnostic applications, we emphasize immune-relevant EV cargoes, such as EV-PD-L1, cytokines, immunomodulatory RNAs, tumor antigens, and immune-cell markers. We further discuss how tumor- and immune-cell-derived EVs reflect immune evasion, checkpoint signaling, immunotherapy response, and resistance, thereby providing an immuno-oncology-oriented framework for patient stratification and treatment monitoring.

However, clinical translation is not merely hindered by technical difficulty—it is systematically obstructed by the interplay between EVs heterogeneity and methodological bias (11, 12). This review uniquely deconstructs this interplay by comparing how different isolation, characterization, and detection strategies select for different EV subpopulations, directly generating the contradictory biomarker results seen across studies. Building on this critical comparison, we identify the most salient unresolved questions and underexplored frontiers—including single-vesicle multi-omics validation, *in vivo* EV tracking, engineered EV immunogenicity, and interpretable AI for biomarker discovery—as the essential road map for achieving robust, clinically actionable EV diagnostics.

## 2 Biological characteristics of extracellular vesicles and their theoretical basis as biomarkers

### 2.1 EVs heterogeneity: biogenesis, size, and molecular cargo

**Figure 1:**
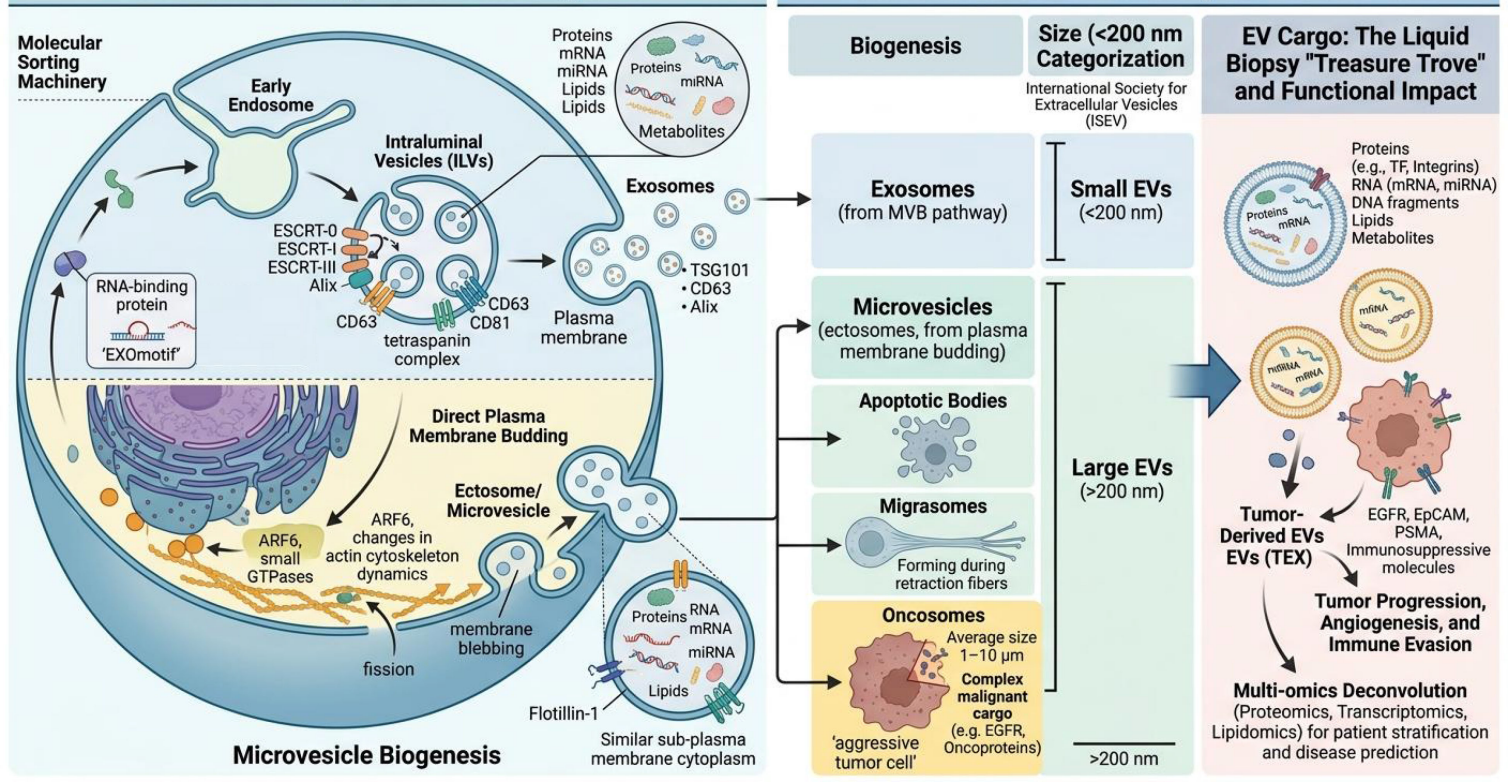
Cellular Origin and Selective Biogenesis of EVs. EVs are heterogeneous membrane-enclosed particles with diverse origins and biogenesis. Exosomes arise from intraluminal vesicles within multivesicular bodies and are released upon fusion with the plasma membrane, whereas microvesicles/ectosomes bud directly from the plasma membrane; large tumor-derived oncosomes represent a specialized microvesicle subtype. Apoptotic bodies are released during apoptosis. EV cargo is selectively packaged through sorting machinery and RNA-binding proteins, reflecting the status of parental cells. ISEV commonly classifies EVs by size into small EVs (<200 nm) and large EVs (>200 nm).

EVs represent a highly heterogeneous population of membrane-enclosed particles secreted by virtually all cell types, with their diversity stemming from distinct biogenesis pathways, size ranges, and molecular cargo (13). The primary classification often distinguishes between exosomes, which originate from the endosomal system via the multivesicular body (MVB) pathway, and microvesicles, which are generated by direct outward budding and shedding of the plasma membrane (14). The fundamental distinction in biogenesis serves as a critical factor influencing the initial physical and molecular properties of extracellular vesicles. In addition to these primary categories, other subtypes—including apoptotic bodies and more recently characterized structures such as migrasomes and oncosomes—contribute to the expanding classification of extracellular vesicles, each exhibiting distinct size ranges and mechanisms of formation (15, 16). For instance, oncosomes, predominantly released by aggressive tumor cells, are notably large, ranging from 1 to 10 µm, and exhibit complex cargo reflective of their malignant origin (16, 17). Currently, no single marker can distinguish between different types of EVs. Therefore, the International Society for Extracellular Vesicles categorizes EVs based on their size: Small EVs <200 nm in diameter and large EVs (including microvesicles [MVs] and apoptotic bodies) >200 nm in diameter (18, 19) (**Figure 1**). This inherent heterogeneity, encompassing variations in size, density, and membrane composition, presents a significant analytical challenge but is crucial for understanding their specific biological functions and for developing precise isolation strategies for liquid biopsy applications (13, 20). The molecular cargo packaged within EVs is not random but is selectively loaded and tightly regulated by the physiological or pathological state of the parent cell (21). This cargo includes a diverse array of biomolecules such as proteins, various RNA species (mRNA, miRNA, non-coding RNAs), DNA fragments, lipids, and metabolites (8). The loading mechanisms involve specific RNA-binding proteins, EXOmotif sequences in miRNAs, and other sorting machinery that ensure the molecular signature of the EV mirrors that of its cell of origin (22). For example, in cancer, tumor-derived EVs (TEX) are enriched with oncogenic proteins (e.g., EGFR), tumor-associated antigens (e.g., EpCAM, PSMA), immunosuppressive molecules, and pro-angiogenic factors, which collectively facilitate tumor progression and immune evasion (23–25). This specificity transforms EVs, particularly those from diseased cells, into a rich “treasure trove” for liquid biopsy, as their cargo provides a snapshot of the originating cell ‘s state (26). Advanced multi-omics approaches-integrating proteomics, transcriptomics, and lipidomics-are now being employed to deconvolute this heterogeneity, revealing cancer-specific protein profiles and distinct RNA cargo that can stratify patients and predict disease progression (27, 28). Therefore, an accurate delineation of EV subpopulations, considering their biogenetic origins, dimensional attributes, and molecular cargo, is essential for harnessing their complete potential as precise and informative biomarkers within clinical diagnostic applications.

### 2.2 Functional roles of EVs in disease pathogenesis and biomarker association

**Figure 2:**
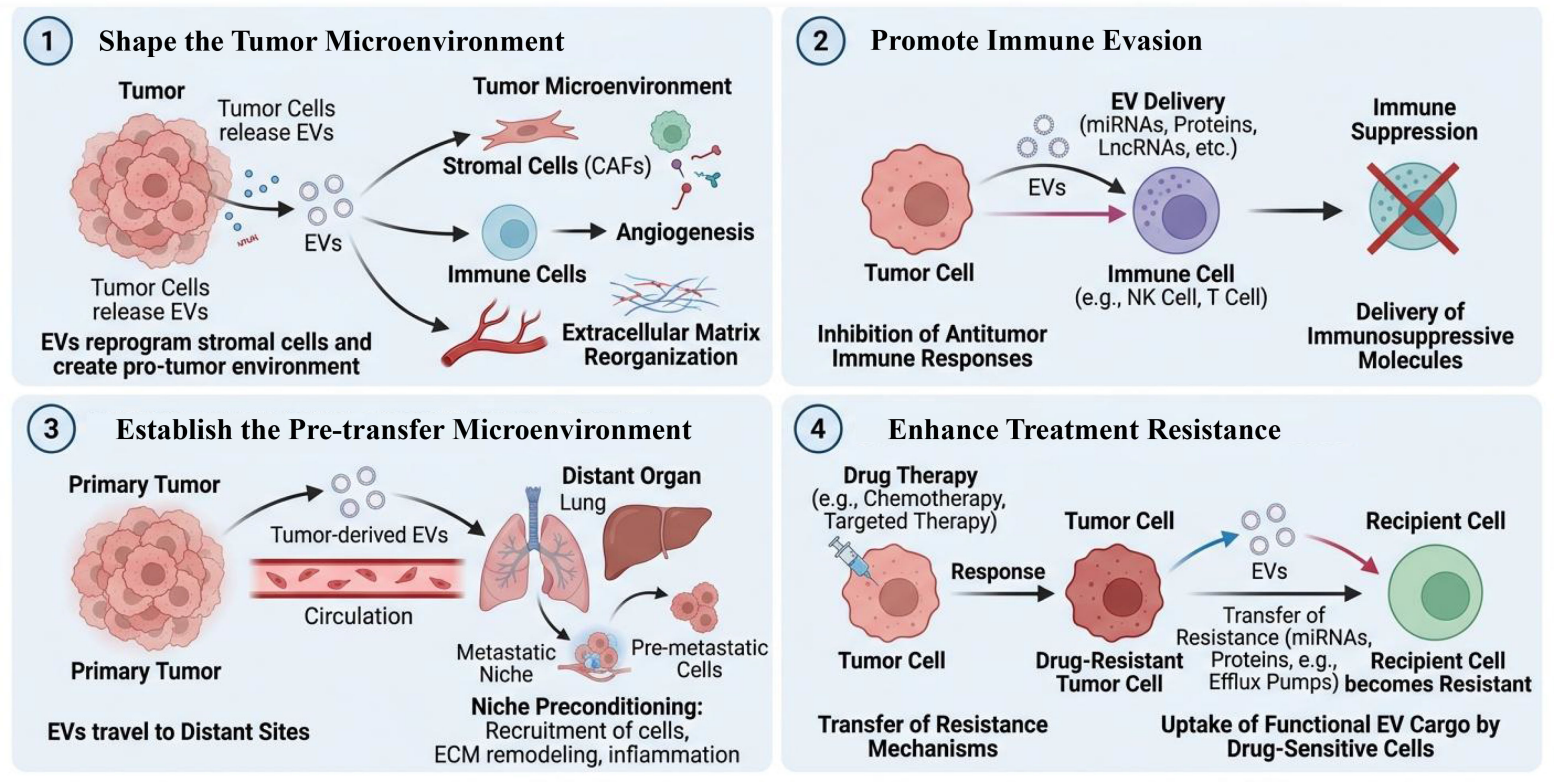
Tumour-derived EVs coordinate key aspects of tumour progression. Tumour cells release heterogeneous EVs that serve as functional packages to reprogram the local and systemic environments. This schematic illustrates that the complex molecular cargo of EVs, including nucleic acids, proteins, and lipids, mediates four critical processes: (1) sculpting the supportive tumor microenvironment, (2) evading immune surveillance, (3) facilitating pre-metastatic niche formation, and (4) promoting therapeutic resistance.

EVs function not only as passive transporters but also as active facilitators of intercellular communication. They assume critical and dynamic roles in the pathogenesis of numerous diseases, with their quantity and molecular cargo closely associated with disease progression. Consequently, EVs represent promising candidates for biomarker development (29). In oncology, tumor-derived EVs are instrumental in shaping the tumor microenvironment, promoting immune escape, establishing pre-metastatic niches, and conferring therapy resistance (23, 24) (**Figure 2**). They achieve this by delivering functional oncoproteins, miRNAs, and other regulatory molecules to recipient cells, including stromal cells, endothelial cells, and immune cells, thereby reprogramming them to support tumor growth and metastasis (30, 31). For instance, platelet-derived extracellular vesicles (PDEVs) can transfer cargo that promotes cancer cell proliferation and metastasis formation (31).The molecular profile of these EVs, such as the ratio of immunostimulatory to immunosuppressive proteins they carry, dynamically changes with disease stage and has been shown to correlate with patient outcomes and responses to immunotherapy (24). In neurological disorders, EVs offer a unique diagnostic opportunity because they can cross the blood-brain barrier (BBB), carrying brain-specific molecules from the central nervous system into the peripheral circulation (14). This capability is particularly valuable for conditions like glioblastoma or HIV-associated neurocognitive disorders, where EVs in biofluids contain neuroinflammatory markers, pathogenic proteins, or viral components that reflect intracranial pathology, enabling minimally invasive “liquid biopsies” for brain tumors and neurodegenerative diseases (32). Similarly, in cardiovascular diseases, EVs derived from endothelial cells, cardiomyocytes, and platelets serve as sensitive reporters of vascular health (33). Their release and cargo are altered in response to endothelial dysfunction, vascular inflammation, atherosclerotic plaque instability, and myocardial injury (33). Specific protein signatures in EVs from paraquat-exposed human brain microvascular endothelial cells, for example, have been linked to toxicity pathways, illustrating how EV cargo mirrors cellular stress (34). Thus, the quantitative and qualitative analysis of EV populations in biofluids provides a window into ongoing pathological processes. The functional roles EVs play-whether in facilitating tumor progression, mediating neuroinflammation, or reflecting cardiovascular damage-are intrinsically tied to their biomarker potential. Their content dynamically shifts with disease severity, making them promising tools for early detection, prognostic stratification, and monitoring therapeutic responses across a broad spectrum of conditions, from cancer and neurodegeneration to cardiovascular and metabolic syndromes (35).

## 3 Biomarker discovery and application of EVs-based liquid biopsy in major diseases

### 3.1 Tumor diagnosis and molecular subtyping

Solid Tumors: EVs have emerged as powerful liquid biopsy tools for the diagnosis and molecular subtyping of solid tumors, offering a non-invasive window into tumor biology. In prostate cancer, EVs isolated from urine and plasma carry tumor-specific molecular cargo that can distinguish malignant from benign conditions. Proteomic profiling of EVs from prostate cancer cell lines representing diverse clinical subtypes, including androgen receptor-positive (AR+), AR-negative/neuroendocrine-positive (AR-/NE+), and AR-negative/neuroendocrine-negative (AR-/NE-) models, reveals distinct protein signatures that cluster by tumor subtype (36). EVs from AR+ cells are enriched in proteins regulated by AR and mTOR signaling, while those from AR-/NE+ cells contain established neuroendocrine markers like SYP and CHGA, and EVs from AR-/NE- models show enrichment in basal cell markers and proteins regulating epithelial-to-mesenchymal transition (36). This proteomic distinction underscores the potential of EV analysis for non-invasive molecular subtyping, which is crucial for prognostication and treatment decisions, especially for aggressive subtypes like AR-/NE+ prostate cancers (36). Similarly, in breast cancer, EVs provide a platform for molecular subtyping. An EV microfluidic affinity purification chip enables the orthogonal isolation of EV subpopulations based on surface markers like epithelial cell adhesion molecule (EpCAM, epithelial origin) and fibroblast activation protein α (FAPα, mesenchymal origin) from patient plasma (37). Profiling of exosomal mRNA (exo-mRNA) from these EV subsets using a subset of PAM50 genes generated 100% concordance with molecular subtypes determined from matched tumor tissue, demonstrating the utility of EV-based liquid biopsy for accurate breast cancer subtyping (37). Furthermore, the protein, metabolite, and fatty acid content of small EVs derived from glioblastoma stem-like cells (GSCs) varies between proneural and mesenchymal subtypes, suggesting a role for EVs in mediating molecular collaboration and contributing to tumor heterogeneity (38). Beyond proteins and RNA, the analysis of circulating EV long RNA profiles from plasma has been used to categorize pancreatic ductal adenocarcinoma (PDAC) into molecular subtypes with distinct prognoses and immunogenic features, offering a tissue-agnostic approach for personalized treatment planning (39). These studies collectively highlight that EVs encapsulate a rich, cell-of-origin molecular signature. The development of cross-species proteomic methods to quantify the human tumor-derived proteome in plasma EVs from patient-derived xenografts further confirms that machine learning can accurately classify the underlying tumor type based on EV protein cargo (40). Therefore, EVs serve as multifaceted biomarkers, enabling not only the detection of solid tumors but also their precise molecular characterization, which is fundamental for implementing precision oncology strategies.

Despite these promising findings, the evidence remains largely exploratory. Many studies rely on cell-line-derived EVs, small patient cohorts, or single-center retrospective samples, which may inflate diagnostic or subtyping accuracy. Reported concordance between EV-based and tissue-based classifications also requires cautious interpretation because independent external validation is often limited. In addition, different EV isolation platforms, including ultracentrifugation, size-exclusion chromatography, precipitation, and immunoaffinity capture, may enrich different EV subsets and co-isolated contaminants, making cross-study comparison difficult. Therefore, although EVs show strong potential for non-invasive tumor subtyping, their robustness across heterogeneous clinical populations remains to be established.

Early Diagnosis and Screening: The pursuit of early cancer detection, particularly for malignancies like PDAC where early diagnosis dramatically improves outcomes, is a major focus of EV-based liquid biopsy research. The rationale stems from the ability of EVs to be released from tumor cells into proximal biofluids, potentially offering a higher concentration of tumor-derived biomarkers compared to peripheral blood, thus enhancing sensitivity for early lesions. For PDAC, biofluids in direct contact with the pancreas, such as pancreatic juice and bile obtained endoscopically, are promising sources for EV enrichment. Studies highlight the role of sEVs, including exosomes, in PDAC pathobiology, carrying oncogenic cargo that regulates tumor progression (41). The molecular analysis of these proximal fluid-derived EVs can reveal early pathogenic events. For instance, a multi-ethnic radiogenomics study integrated imaging features with plasma-derived EV microRNA (miRNA) profiles and identified a signature of three low-abundance EV miRNAs that robustly distinguished malignant from benign pancreatic lesions, facilitating early diagnosis (accuracy=0.894, and AUC = 0.897) (42). This approach underscores the potential of combining EV biomarkers with other clinical data for enhanced detection. Furthermore, the analysis of circulating EV long RNA from plasma has been used not only for subtyping but also implies utility in early detection by reflecting the tumor’s transcriptomic state (39). The concept of spatial enrichment is critical; EVs released from early-stage tumors might be more concentrated in locally draining fluids before disseminating broadly into the systemic circulation. This principle is explored beyond PDAC in other hard-to-detect cancers. In brain tumors, for example, cerebrospinal fluid (CSF), due to its direct contact with the central nervous system, is a more enriched source of tumor-derived EVs and other biomarkers like ctDNA compared to peripheral blood, offering superior sensitivity for diagnosis and monitoring (43). Similarly, in bladder cancer, urine is a direct and rich source of tumor-derived EVs. Research indicates that urinary EVs (uEVs) can reflect the molecular subtypes of bladder cancer, and an AI-driven pipeline analyzing uEV transcriptomes shows promise for high-precision subtyping and prognosis, leveraging the spatial advantage of urine sampling (44). These approaches address the limitation of low biomarker abundance in peripheral blood for early-stage disease. The technical advancement in EV isolation and analysis, such as the use of size-exclusion chromatography for uEVs or sensitive sequencing for circulating EV long RNA (exLR), is key to unlocking this potential (45). Therefore, targeting EVs from biofluids that are anatomically proximal to the tumor site represents a strategic avenue to improve the sensitivity of liquid biopsies for early cancer diagnosis and screening, moving beyond the limitations of peripheral blood sampling alone.

However, early detection requires particularly high specificity, and this remains a major limitation of current EV studies. Many candidate EV biomarkers have been identified in retrospective discovery cohorts with small numbers of early-stage cases and limited inclusion of benign mimics. EV miRNAs, long RNAs, or proteins may also be altered by inflammation, fibrosis, obstruction, tissue injury, or premalignant lesions, reducing cancer specificity. Moreover, negative or less successful findings are likely underreported, and few studies have tested whether EV assays outperform established markers or imaging in prospective screening settings. Thus, multicenter validation in high-risk and benign-control cohorts is essential.

Molecular Subtyping: Molecular subtyping via EV analysis provides a minimally invasive method to guide precision therapy by revealing critical tumor characteristics such as driver mutations and immune profiles. Transcriptomic and proteomic profiling of EVs can accurately reflect the molecular subtype of the parent tumor, enabling treatment decisions without the need for repeated tissue biopsies. In lung cancer, EVs can be used to identify actionable mutations and define subtypes. For small cell lung cancer (SCLC), a highly heterogeneous disease, measuring the mRNA expression of subtype-defining transcription factors (ASCL1, POU2F3, NEUROD1) in circulating exosome-rich EVs allows for accurate identification of SCLC molecular subtypes, which is crucial as different subtypes may have differential therapeutic vulnerabilities (46). Furthermore, in non-small cell lung cancer (NSCLC), the detection of specific mutations like mesenchymal-epithelial transition exon 14 (METex14) skipping from liquid biopsy, which can include EV analysis, is essential for initiating targeted therapy with MET inhibitors (47). Beyond mutations, EV cargo analysis facilitates comprehensive molecular subtyping across various cancers. In colorectal cancer (CRC), transcriptomic profiling of RNA within plasma extracellular vesicles (evRNA) combined with deep learning algorithms can reliably predict consensus molecular subtypes (CMS), allowing for longitudinal monitoring of subtype changes under treatment pressure (48). Similarly, in diffuse large B-cell lymphoma (DLBCL), proteomic analysis of secreted EVs from cell lines representing germinal center B-cell (GCB) and activated B-cell (ABC) subtypes demonstrates that the EV proteome possesses the capacity to separate these molecular subtypes, suggesting EVs as a tool for patient diagnosis and follow-up (49). EV-based subtyping also extends to defining immunogenic characteristics. For PDAC, exLR profiling not only categorizes tumors into subtypes but also reveals signatures predictive of immunogenic features and clinical outcomes, including concordance of immunoregulators between tissue and blood EVs (39). This is particularly relevant for immunotherapy, where understanding the tumor immune microenvironment is key. EVs themselves play a role in modulating this environment; tumor-derived EVs can carry immune checkpoint molecules like PD-L1, contributing to immunosuppression (50). Analyzing EVs for such cargo could non-invasively inform immunotherapy strategies. The process relies on advanced analytical techniques. For example, a shotgun proteomics approach on EVs from prostate cancer cell lines identified nearly 4000 proteins, enabling the derivation of classic AR or NE identity gene signatures relevant for prognostication. In breast cancer, molecular subtyping using EV mRNA via the exo-PAM50 test shows perfect concordance with tissue-based subtyping (37). These examples underscore that EVs serve as circulating surrogates of the tumor, encapsulating the genomic, transcriptomic, and proteomic information necessary for precise molecular subtyping, thereby directly informing the selection of targeted therapies and immunotherapies in a dynamic and non-invasive manner.

Nevertheless, EV-based molecular subtyping faces important interpretive challenges. Bulk EV profiles contain signals from tumor cells as well as immune, stromal, endothelial, platelet, and normal epithelial cells, making tumor-specific attribution difficult. Subtype signatures may also be influenced by tumor burden, systemic inflammation, prior treatment, and biofluid source. For mutation or transcriptomic classification, low tumor-derived EV fractions can limit sensitivity, especially in early-stage or low-shedding tumors. In addition, AI-based classifiers trained on small cohorts may overfit and perform less well in external populations. Standardized workflows and prospective validation against tissue and clinical outcomes are therefore required.

### 3.2 Disease progression monitoring and prognostic assessment

Treatment Response Monitoring: EVs serve as dynamic, real-time biomarkers for monitoring response to cancer therapy, particularly immunotherapies, by reflecting changes in the tumor and its microenvironment. A key mechanism of immune evasion involves the expression of immune checkpoint proteins like programmed death-ligand 1 (PD-L1) on tumor cells and their secreted EVs. Tumor-derived EVs carrying PD-L1 can directly suppress T-cell activity, contributing to an immunosuppressive tumor microenvironment (50, 51). Consequently, the level of PD-L1 on circulating EVs has emerged as a promising pharmacodynamic biomarker. In patients receiving immune checkpoint inhibitors (ICIs) for cancers such as melanoma or non-small cell lung cancer (NSCLC), longitudinal monitoring of EV-associated PD-L1 could provide insights into therapeutic efficacy. A decrease in PD-L1-positive EVs may indicate successful blockade of the PD-1/PD-L1 axis and immune reactivation, whereas persistent or increasing levels might suggest primary or acquired resistance. EVs mediate complex communication within the tumor immune microenvironment (TIME). They are involved in remodeling the TIME, and alterations in their quantity and composition can influence immunotherapy efficacy (51). For example, tumor-derived EVs can “cold” tumors by promoting immunosuppression and immune evasion, thereby contributing to resistance against ICIs (51). Monitoring specific EV cargo beyond PD-L1, such as non-coding RNAs or other immunomodulatory proteins, could offer a more comprehensive view of the evolving immune landscape under treatment pressure. The potential of EVs in this context is underscored by their role in anti-tumor immunity and as mediators of communication between cancer and immune cells (52, 53). Research into EV-based strategies for tumor immunotherapy highlights their application in modulating immune responses and overcoming tumor-induced tolerance (54). From a technical perspective, advancements in EV isolation and characterization are broadening our understanding of their functions and enhancing their utility as biomarkers for monitoring treatment response (55). While the cited references primarily establish the foundational role of EVs in immune modulation and their potential as therapeutic agents or targets, they strongly support the conceptual framework that EV cargo, including immune checkpoint molecules, is dynamically regulated by therapy. Therefore, serial analysis of EVs from liquid biopsies presents a minimally invasive strategy to track molecular adaptations of the tumor during treatment, enabling early detection of response or resistance, and potentially guiding timely therapeutic adjustments.

Minimal Residual Disease and Recurrence Warning: The detection of minimal residual disease (MRD) and early recurrence is a critical unmet need in oncology, particularly for aggressive cancers like glioblastoma (GBM) where imaging can be difficult to interpret. Extracellular vesicles derived from tumor cells offer a promising solution for early warning, as they are shed into biofluids continuously and carry molecular signatures of the residual tumor cells. In GBM, EVs are crucial mediators within the tumor microenvironment, facilitating communication that supports tumor proliferation, invasion, and immune evasion (56). This active secretion makes EVs a rich source of biomarkers. Studies highlight the potential of liquid biopsy, including EV analysis, for monitoring primary brain tumors (57). For pediatric brain tumors, which share similar challenges, liquid biopsy of blood, urine, and CSF to sample cfDNA, extracellular vesicles, and tumor-associated proteins is being developed for molecular diagnosis and treatment response monitoring (58). Specifically for GBM, tumor-derived biomarkers in liquid biopsy, which include components carried by EVs such as nucleic acids and proteins, are a focus for detecting recurrence (59). While the provided references do not explicitly list miR-21, GFAP, or CD44 in EVs for GBM recurrence, they establish the strong rationale for EV-based MRD detection. EVs released by GBM cells contain biomolecules that mirror the tumor’s state. For instance, EVs derived from temozolomide-treated and non-treated GBM cells show distinct molecular profiles and can modulate macrophage responses, indicating their dynamic nature based on treatment status (60). Furthermore, large EVs released by GBM cells have been shown to drive tumor invasiveness via mechanisms involving Connexin 43, highlighting their active role in progression (61). The heterogeneity of EVs, as revealed by single EV analysis technologies, indicates that specific EV subpopulations with unique molecular signatures could reflect the status of parental cancer cells during progression or therapy, supporting their use for in-depth monitoring via liquid biopsy (62). In the broader context of liquid biopsy for brain tumors, CSF is often a more enriched source of tumor-derived biomarkers than plasma due to proximity to the tumor site (43). The analysis of ctDNA in CSF for glioma molecular subtyping demonstrates the feasibility of using proximal biofluids for tumor profiling (63). By extension, EVs in CSF or even urine-considered a “liquid gold” source for less invasive monitoring-could harbor early molecular signs of recurrence, such as specific miRNAs, circRNAs, or proteins, potentially preceding radiographic changes. Therefore, leveraging the molecular cargo of EVs from accessible biofluids provides a promising strategy for the early detection of MRD and recurrence, enabling earlier clinical intervention.

Prognostic Biomarkers: EVs carry molecular cargo that reflects tumor aggressiveness and patient outcome, making them valuable sources of prognostic biomarkers. In NSCLC and other malignancies, specific EV-contained molecules are associated with disease stage, metastatic potential, and survival. While the provided references do not specifically mention miR-124 in NSCLC EVs, they extensively discuss the role of EVs in tumor progression and their potential as prognostic tools. Tumor-derived EVs are multifunctional entities in the tumor microenvironment (TME), influencing disease progression, and their number and content can vary depending on disease stage or response to therapy, raising the exciting possibility of their use for risk stratification and prognostic purposes (64). For instance, in PDAC, exLR profiling not only enables subtyping but also provides signatures for predicting clinical outcomes (39). The exLR-based subtyping of PDAC categorizes patients into groups with distinct prognoses, directly linking EV molecular profiles to survival (39). Similarly, in breast cancer, an AI-driven uEV liquid biopsy pipeline was used to develop a prognostic score that stratified bladder cancer patients into risk groups based on survival outcomes, demonstrating the prognostic utility of EV transcriptomes (44). The prognostic value is rooted in the biological functions of EVs. They are key regulators of tumor neovasculature and mediate immunosuppression, both processes directly linked to aggressive disease and poor prognosis (65). In glioblastoma, EVs induce mesenchymal transition in proneural cells via NF-κB/STAT3 signaling, a shift associated with increased aggressiveness and therapeutic resistance, which could be captured prognostically by analyzing relevant EV cargo (66). Furthermore, the dynamic heterogeneity of single EVs monitored during cancer progression and therapy suggests that specific EV signatures could indicate tumor status and aggressiveness (62). From a technical standpoint, advances in disentangling the complexity of tumor-derived EVs enhance our ability to establish their utility as cancer biomarkers, including for prognosis (55). Although the specific example of miR-124 in NSCLC EVs is not covered, the established principles strongly support that EV cargo, including miRNAs, proteins, and RNAs, can correlate with disease stage and recurrence risk. The analysis of such EV-derived molecules from liquid biopsies offers a non-invasive means to generate prognostic information, aiding in patient stratification and personalized management strategies.

### 3.3 Exploration of EV biomarkers in neurological and cardiovascular diseases

Glioblastoma (GBM) presents significant challenges for monitoring due to the invasiveness of tissue biopsy and the limitations of neuroimaging. Extracellular vesicles in biofluids offer a promising alternative for non-invasive monitoring. While CSF is a direct source, its collection is invasive. Consequently, urine has emerged as an attractive, easily accessible “liquid gold” source for EV-based liquid biopsy in GBM and other brain tumors. The rationale is that EVs released from brain tumors may cross the blood-brain barrier or be cleared through the renal system, allowing tumor-derived molecules to be detected in urine. Research in pediatric brain tumors highlights the use of liquid biopsy from blood, urine, and CSF to sample biomarkers including extracellular vesicles for molecular diagnosis and treatment response monitoring (59). Although the provided references do not detail specific urinary EV proteomic signatures for GBM recurrence, they establish the strong foundation for EV-based monitoring in brain tumors. Liquid biopsy for primary brain tumors, utilizing circulating tumor cells, cell-free nucleic acids, extracellular vesicles, and miRNA, is an area of active development to improve clinical management (57). The role of liquid biopsy in detecting molecular tumor biomarkers in GBM patients is evaluated, acknowledging the challenge posed by the blood-brain barrier but also noting advances in the field (67). EVs are crucial mediators in the GBM tumor microenvironment, involved in processes like proliferation, immune evasion, and invasion (56). Their release is a dynamic process, and their molecular cargo reflects the state of the parent tumor cells. For instance, EVs derived from temozolomide-treated and non-treated GBM cells show distinct molecular profiles, indicating that treatment pressure alters EV signatures, which could be monitored serially (60). Furthermore, the heterogeneity of EVs, as monitored by single EV analysis, reveals complex distributions affected by oncogenic transformation and therapy, suggesting that precise molecular profiling of circulating single EVs could enable non-invasive diagnostic monitoring (62). The exploration of urinary EVs for GBM leverages the principle that biofluids accessible for frequent sampling can provide dynamic molecular information. In the broader context of liquid biopsy for brain tumors, targeted approaches, such as sampling blood from tumor-draining veins or using CSF, are hypothesized to enhance biomarker detection sensitivity compared to peripheral blood (43). By extension, deep proteomic or other omics analyses of urinary EVs could potentially uncover specific protein signatures correlating with tumor burden, treatment response, or early recurrence, providing a completely non-invasive tool for frequent patient monitoring. This approach aligns with the ongoing efforts to develop liquid biopsy strategies that supplement imaging for better management of glioblastoma.

However, EV-based monitoring in GBM remains largely exploratory. Most studies are small, single-center, retrospective, and lack independent validation. CSF is enriched for brain tumor-derived EVs but is invasive, whereas blood and urine are easier to collect but contain low and variable tumor-derived EV fractions. In particular, evidence supporting urinary EVs for GBM recurrence or treatment monitoring remains limited. EV signals may also be confounded by neuroinflammation, therapy-induced injury, steroid use, and differences in EV isolation methods. Therefore, prospective longitudinal studies with standardized workflows and matched imaging or clinical endpoints are needed.

EVs play significant roles in cardiovascular pathophysiology, with endothelial microparticles (EMPs), a subtype of EVs released from activated or apoptotic endothelial cells, serving as key biomarkers and mediators of vascular disease. While the provided references focus primarily on cancer, the conceptual parallels in EV biology are informative. In cardiovascular diseases, EMPs carry bioactive molecules from their parent endothelial cells, reflecting endothelial health. Elevated levels of EMPs and their specific cargo, such as tissue factor (a potent initiator of the coagulation cascade) and inflammatory cytokines, are indicative of vascular endothelial dysfunction, a critical early event in atherosclerosis and thrombotic disorders. This makes EMPs sensitive circulating biomarkers for assessing the risk of acute coronary syndrome (ACS) and other thrombotic events. Although not explicitly covered in the cancer-centric references, the general principles of EV-mediated communication apply. EVs are mediators of intercellular communication, carrying proteins, lipids, and nucleic acids that can alter recipient cell phenotype (68). In the context of a pro-thrombotic and inflammatory state akin to the tumor microenvironment, EVs from activated cells could promote pathological processes. For instance, in cancer, tumor-derived EVs are involved in promoting angiogenesis and modulating the immune response, processes that also have parallels in cardiovascular inflammation and plaque instability (65). Furthermore, the role of EVs in immunosuppression and shaping the tumor microenvironment suggests that in cardiovascular disease, EVs might similarly modulate local inflammatory and reparative responses within the vascular wall (50). The assessment of EV number and composition is emerging as a tool in various pathological conditions. The references discuss the potential of EVs in tumor diagnosis, highlighting their use as biomarkers due to changes in their structure and cargo during tumor initiation and progression (68). This diagnostic utility can be extrapolated to cardiovascular diseases, where changes in EMP profiles could signal endothelial injury or activation. Moreover, the discussion on EV-based strategies for immunotherapy underscores the potential to target EV pathways for therapeutic benefit, a concept that could translate to modulating EV release or function to mitigate endothelial dysfunction and thrombosis (54). Therefore, while the specific literature on EMPs in ACS is not included here, the established biology of EVs as carriers of cell-state-specific information and mediators of intercellular signaling strongly supports their role as sensitive indicators of endothelial health and thrombotic risk in cardiovascular diseases, holding promise for risk stratification and early intervention.

Nevertheless, EMPs and other EV subsets are not yet established cardiovascular biomarkers. Reported associations with endothelial dysfunction, thrombosis, or ACS vary across studies because EV quantification is highly affected by sample handling, centrifugation, storage, flow cytometry thresholds, and marker selection. EMP levels may also be confounded by platelet-or leukocyte-derived EVs, age, diabetes, hypertension, renal dysfunction, infection, smoking, and medication use. Many studies remain small, observational, and single-center, with limited evidence of added value beyond established biomarkers or imaging. Larger prospective studies with standardized EV phenotyping are therefore required.

### 3.4 EV biomarkers in cancer immunotherapy

Cancer immunotherapy, particularly immune checkpoint blockade targeting the PD-1/PD-L1, has reshaped the treatment landscape of multiple malignancies. However, durable responses occur only in a subset of patients, and current predictive biomarkers, including tissue PD-L1 immunohistochemistry (IHC), tumor mutational burden (TMB), microsatellite instability, and T cell-inflamed gene expression signatures, have important limitations related to spatial heterogeneity, temporal variability, sampling bias, and assay inconsistency (69–72). EVs provide a complementary liquid biopsy approach because they can capture dynamic tumor- and immune-cell-derived molecular information from the circulation or other biofluids. In the context of immunotherapy, EV biomarkers are particularly relevant because EVs not only reflect the immune status of the tumor microenvironment but may also actively participate in immune suppression, immune activation, and treatment resistance.

EV-associated PD-L1 is one of the most extensively studied immunotherapy-related EV biomarkers. Tumor cells can release EVs carrying functional PD-L1 on their surface, and these PD-L1-positive EVs can suppress T-cell activation, inhibit cytokine production, and promote systemic immune evasion (73, 74). Mechanistically, EV PD-L1 may act beyond the local tumor site by circulating through peripheral blood and interacting with PD-1-positive T cells, thereby extending checkpoint-mediated immunosuppression to distant immune compartments. Preclinical studies have shown that suppression of EV PD-L1 release can enhance systemic antitumor immunity and improve the efficacy of immune checkpoint blockade (74). Clinically, elevated levels of circulating EV PD-L1 have been reported in several cancers and have been associated with disease progression, immune dysfunction, and variable responses to anti-PD-1/PD-L1 therapy (75–77). Importantly, serial monitoring of EV PD-L1 may provide pharmacodynamic information during treatment. For example, changes in circulating exosomal PD-L1 during early treatment have been associated with clinical outcome in melanoma and other solid tumors, suggesting that EV PD-L1 may help distinguish responders from non-responders before conventional radiographic assessment.

Beyond PD-L1, EVs carry diverse immune-modulatory cargo that may influence the tumor immune microenvironment and provide additional biomarker information. Tumor-derived EVs may contain immunosuppressive proteins, including transforming growth factor-β, Fas ligand, TRAIL, CD39/CD73-related adenosine pathway components, NKG2D ligands, and other molecules capable of impairing cytotoxic T cells, natural killer cells, dendritic cells, or antigen-presenting functions (78, 79). EVs also transport immune-regulatory nucleic acids, including miRNAs, long non-coding RNAs, circular RNAs, and other RNA species that can modulate macrophage polarization, myeloid-derived suppressor cell expansion, regulatory T-cell induction, antigen presentation, and interferon signaling (78–80). Therefore, EV-based immunotherapy biomarkers should not be restricted to PD-L1 alone. Integrated EV protein and RNA signatures may provide a broader picture of tumor-immune interactions, including immune activation, immune exclusion, myeloid inflammation, and adaptive immune resistance.

EV signatures may also help characterize “hot” and “cold” tumor immune phenotypes. Immunologically “hot” tumors are typically enriched in cytotoxic T-cell infiltration, interferon-γ signaling, antigen presentation machinery, chemokine expression, and immune checkpoint upregulation, whereas “cold” tumors are characterized by poor T-cell infiltration, defective antigen presentation, stromal exclusion, myeloid suppression, or low inflammatory signaling (81). Because tissue biopsy captures only a restricted tumor region, it may fail to represent spatially heterogeneous immune states across primary and metastatic lesions. EVs may partially overcome this limitation by sampling molecular signals shed from multiple tumor and immune compartments. For example, EV cargo reflecting interferon signaling, antigen presentation, T-cell recruitment, myeloid suppression, or checkpoint activation could potentially complement tissue-based immune profiling and provide a longitudinal readout of transitions between immune-inflamed, immune-excluded, and immune-desert phenotypes during therapy.

EV-based biomarkers are best viewed as complementary rather than replacements for established immunotherapy biomarkers. PD-L1 IHC remains clinically useful but is affected by antibody clone, scoring system, tumor heterogeneity, inducible expression, and differences between archival and contemporary tissue samples (71). TMB can capture neoantigen potential but does not directly measure antigen presentation, immune infiltration, or immune suppression, and its predictive value varies across tumor types (72). T cell-inflamed gene expression profiles reflect an adaptive immune response and may identify tumors more likely to respond to PD-1 blockade, but they generally require adequate tumor tissue and may not be feasible for repeated monitoring. In this context, EV biomarkers could provide several advantages: they are minimally invasive, suitable for serial sampling, capable of capturing both tumor-derived and host immune-derived information, and potentially informative for dynamic resistance mechanisms. A combined biomarker framework integrating EV PD-L1, EV immune-regulatory RNA/protein signatures, tissue PD-L1 IHC, TMB, and T cell-inflamed gene signatures may therefore improve patient stratification, early response assessment, and detection of acquired resistance.

Despite this promise, EV biomarkers in cancer immunotherapy remain insufficiently standardized for routine clinical use. Major challenges include the lack of uniform methods for isolating tumor-derived EVs, variable detection platforms for EV PD-L1, difficulty distinguishing tumor-derived EVs from immune-cell- or platelet-derived EVs, inconsistent normalization strategies, and limited prospective validation in large immunotherapy-treated cohorts. In addition, functional attribution is complex because circulating EV preparations may contain mixtures of vesicles, lipoproteins, protein aggregates, and soluble immune checkpoint proteins. Future studies should combine standardized EV isolation and characterization with single-EV phenotyping, multiplexed immune cargo profiling, matched tissue immune analysis, and longitudinal clinical endpoints. Such efforts will be essential to determine whether EV-based immune signatures can robustly complement existing biomarkers and guide personalized immunotherapy.

## 4 Standardization challenges and clinical translation pathways

### 4.1 Preanalytical variables and standardization initiatives

The translation of EVs from promising research entities into reliable liquid biopsy biomarkers is fundamentally hampered by significant preanalytical variability, which critically affects EV yield, integrity, and molecular composition. Sample collection and handling procedures are primary sources of this irreproducibility. For blood-based EV analysis, the choice of anticoagulant in collection tubes (e.g., K3-EDTA, citrate, or specialized cell-free DNA tubes) can influence subsequent EV isolation and characterization (82). The time delay between blood draw and plasma processing, as well as centrifugation speeds and durations used to generate platelet-poor or platelet-free plasma, are well-documented variables that alter EV particle counts and protein content, potentially confounding biomarker measurements (83). For instance, delayed processing can increase the particle number and protein content of microvesicles and introduce leukocyte-derived contaminants, while specialized tubes may stabilize certain EV subpopulations (83). Similarly, for urine EVs, storage temperature significantly impacts the recovery of disease-relevant RNA biomarkers, with storage at -20 °C leading to decreased levels of kidney-associated transcripts compared to -80 °C (84). Furthermore, the EV isolation workflow itself-whether ultracentrifugation, size-exclusion chromatography, density gradient centrifugation, or emerging microfluidic techniques-extracts different EV subpopulations with varying efficiencies and purities, directly affecting downstream molecular profiles (85). This variability, which a survey indicated can reach 94% across different laboratories, is a major obstacle to comparing results between studies and establishing robust clinical assays (82). Recognizing this critical bottleneck, international consortia have launched major standardization efforts. The International Society for Extracellular Vesicles (ISEV) has been instrumental, first with its Minimal Information for Studies of Extracellular Vesicles (MISEV) guidelines and more recently with the minimal information for blood EV research framework, which provides specific recommendations for reporting blood collection, handling, and sample composition to enhance reproducibility (86). Other initiatives, such as the Standard the standard preanalytical code promoted by groups like GEIVEX, aim to codify preanalytical conditions in biobanking, providing a structured way to document variables from collection to storage. The Extracellular Vesicle Flow Cytometry Working Group has developed the MIFlowCyt-EV framework to standardize the reporting of flow cytometry experiments, a common but highly variable method for EV analysis (87). These collective efforts to establish standardized operating procedures and reporting frameworks are essential to control preanalytical conditions, improve data credibility, and pave the way for the validation of EV-derived biomarkers in large patient cohorts (87).

To operationalize the MISEV2023 recommendations in EV biomarker research, several core expectations are particularly relevant. First, studies should provide transparent and complete reporting of preanalytical and analytical variables, including biofluid source, collection tube, anticoagulant, time to processing, centrifugation protocol, storage temperature, freeze–thaw cycles, hemolysis or platelet contamination, EV separation method, input volume, recovery, purity, normalization strategy, and data-analysis workflow. Second, EV identity should not be inferred from a single parameter or a single isolation method. Instead, complementary characterization is required, including particle-based measurements, enrichment of EV-associated protein markers, assessment of morphology or single-particle properties, and evaluation of non-EV co-isolated components. Third, appropriate controls should be incorporated to assess contamination from lipoproteins, protein aggregates, ribonucleoproteins, platelets, leukocytes, or other biofluid-specific confounders, because these entities may carry overlapping RNA or protein signatures and may falsely appear as EV-associated biomarkers. Fourth, consistent nomenclature should be used. Unless vesicles are shown to originate from a specific biogenesis pathway, MISEV2023 recommends using operational terms such as “small EVs,” “large EVs,” or “EV-enriched preparations,” rather than assigning the term “exosomes” solely on the basis of size or a single isolation method. When these criteria are applied to the current biomarker literature, many studies remain limited by incomplete preanalytical reporting, single-method EV isolation, variable normalization strategies, insufficient contamination controls, inconsistent nomenclature, small retrospective cohorts, and limited external validation. Such incomplete adherence to MISEV2023-level expectations directly compromises inter-study reproducibility, weakens confidence in biomarker specificity, and creates barriers to analytical validation and regulatory approval. Thus, MISEV2023 should serve not only as a reporting checklist but also as a quality framework for interpreting existing evidence and designing prospective clinical validation studies. **Table 2** summarizes the application of MISEV2023 principles to EV biomarker research.

**Table 2:** Applying MISEV2023 principles to EV biomarker studies.

MISEV2023-related requirement	Common limitation in current biomarker studies	Impact on clinical translation
Detailed reporting of biofluid collection, anticoagulant, processing time, centrifugation, storage, and freeze–thaw cycles	Preanalytical variables are often incompletely reported	Poor reproducibility and limited cross-study comparability
Assessment of sample quality, including hemolysis and platelet contamination for blood EVs	Platelet activation or cellular contamination may not be controlled	False disease-associated EV signals
Transparent EV isolation workflow with input volume, recovery, purity, and co-isolated contaminants	Single isolation method used without purity or recovery assessment	Biomarkers may derive from non-EV components
Orthogonal EV characterization using particle analysis, protein markers, morphology or single-particle methods	EV identity sometimes inferred from limited markers	Uncertain attribution of cargo to EVs
Use of EV-enriched markers and negative/non-EV contamination markers	Lipoproteins, protein aggregates, and ribonucleoproteins often not assessed	Reduced specificity of EV RNA/protein biomarkers
Standardized flow cytometry reporting according to MIFlowCyt-EV	Variable thresholds, antibody panels, and calibration methods	Inconsistent EV subset quantification
Predefined normalization and statistical analysis plans	Normalization varies by volume, protein, particle number, or RNA input	Inflated or non-reproducible diagnostic accuracy
Independent validation and blinded prospective cohorts	Many studies remain small, single-center, or retrospective	Insufficient evidence for regulatory approval

### 4.2 Barriers to clinical validation and regulatory approval

The path from discovering an EV-associated biomarker to its acceptance as a clinically validated diagnostic tool is constrained not only by regulatory requirements but also by incomplete adherence to MISEV2023-level rigor. In particular, insufficient EV characterization, incomplete reporting of preanalytical variables, inadequate contamination controls, and inconsistent EV nomenclature can make it difficult to determine whether a measured molecular signal truly derives from EVs or from co-isolated non-EV components. These limitations reduce reproducibility across laboratories and cohorts, complicate assay standardization, and weaken the analytical validity required for regulatory review. A candidate biomarker must progress from discovery to validation in large, multicenter, prospective cohorts, but this process is only meaningful when preanalytical handling, EV separation, characterization, normalization, and data analysis are sufficiently standardized and transparently reported. A candidate biomarker, such as α-synuclein in plasma EVs for Parkinson’s disease or specific miRNAs in seminal plasma for azoospermia, must progress from initial discovery in well-controlled but often small-scale studies to validation in large, multi-center, prospective cohort studies (88, 89). These studies must demonstrate robust diagnostic performance, including high sensitivity, specificity, and area under the curve, and establish clear prognostic value or correlation with existing gold-standard methods like imaging or tissue biopsy (88). Despite these challenges, several EV-based technologies have already crossed the translational gap and entered routine clinical use, demonstrating real-world applicability. For instance, the ExoDx Prostate IntelliScore test (Exosome Diagnostics) analyzes a three-gene RNA signature from urine-derived exosomes to stratify men with elevated PSA for the risk of high-grade prostate cancer. It has received Medicare coverage and is integrated into urology clinic workflows in the United States, reducing unnecessary biopsies by over 30% in validation cohorts (90). Similarly, the ExoDx Lung (ALK) test uses plasma EV RNA to detect ALK fusions in non−small cell lung cancer, offering a minimally invasive alternative to tissue biopsy that is already being offered as a laboratory-developed test in several clinical laboratories. In the field of obstetrics, a screen based on placental EV−associated miRNAs has been clinically validated for early prediction of preeclampsia, with a commercial version undergoing large-scale deployment in hospital prenatal care settings (91). These examples illustrate that when coupled with robust standardization—such as adopting standardized collection tubes (e.g., cell−free DNA BCT tubes), automated isolation platforms, and strict quality control according to ISO 15189—EV assays can achieve the reproducibility and throughput required for daily clinical decision-making. This process is immensely complicated by the preanalytical and analytical variabilities discussed previously, as inconsistent sample handling or isolation methods across different clinical sites can invalidate results. For example, the validation of a seminal plasma miRNA biomarker for azoospermia required careful consideration of the sample source (whole plasma vs. isolated EVs) as a preanalytical variable to optimize the clinical assay (89). The second significant obstacle is the clinical applicability of the underlying technology platforms. While research laboratories employ sophisticated techniques like single-vesicle imaging, deep proteomics, or RNA sequencing that offer high performance, these methods often lack the high throughput, automation, low cost, and operational simplicity required for routine clinical laboratory use (92). The ideal clinical platform must be rapid, reproducible, and easily integrated into existing clinical workflows. There is a growing push to develop and validate such platforms, including standardized protocols for EV isolation and quantification that are scalable and maximize the scientific value of precious clinical specimens (93). Finally, navigating the regulatory landscape presents a formidable challenge. To be marketed as an *in vitro* diagnostic (IVD) product, an EV-based test must satisfy stringent requirements from agencies like the U.S. Food and Drug Administration (FDA) or the European Medicines Agency (EMA). This demands comprehensive analytical performance validation (e.g., precision, accuracy, limit of detection), definitive proof of clinical utility, and adherence to quality management systems such as ISO 13485 for manufacturing (94). The current lack of standardization directly contravenes these regulatory needs, as it prevents the consistent production and characterization of EVs as either biomarkers or therapeutic agents (95). Harmonizing general analytical guidelines, like the MIQE guidelines for quantitative PCR, with domain-specific standards like MISEV, is proposed as a scalable blueprint to strengthen the analytical validity and reproducibility essential for regulatory submission (96). Ultimately, overcoming these interconnected barriers-through rigorous validation in standardized settings, the development of clinically viable platforms, and strategic engagement with regulatory science-is imperative for EV-based liquid biopsies to fulfill their transformative potential in precision medicine.

## 5 Emerging detection strategies and technological innovations

Recent advances in EV-based liquid biopsy are moving the field beyond conventional multistep workflows that rely on separate EV isolation, cargo extraction, and molecular detection. Two complementary directions are particularly important: separation-free or integrated detection platforms, and spatial liquid biopsy strategies based on near-source biofluids. Together, these approaches aim to improve analytical sensitivity, reduce sample loss, preserve EV-associated molecular information, and enhance clinical feasibility.

### 5.1 Separation-free and integrated EV detection platforms

Separation-free EV detection technologies are designed to identify target EVs or EV-associated cargo directly in complex biofluids, thereby minimizing losses and bias introduced by conventional isolation procedures. These methods are particularly attractive for clinical implementation because they simplify workflow, shorten turnaround time, and may facilitate point-of-care testing. Representative strategies include liposome-assisted EV fusion, nucleic acid amplification-based assays, CRISPR-enabled detection, and one-pot molecular profiling platforms.

Liposome-assisted or fusion-based assays enable detection reagents to access EV cargo without extensive purification or conventional RNA extraction. For example, the EV alarm platform uses anionic liposomes as membrane-fusion carriers to deliver probes for multiplex EV-miRNA detection directly from plasma, achieving sensitivity comparable to quantitative PCR with a simplified workflow (97). Similarly, extraction-free CRISPR-based systems, such as opiCRISPR, combine immunorecognition, recombinase polymerase amplification, and CRISPR-Cas13a detection for ultrasensitive analysis of EV surface proteins such as Glypican-3, showing potential utility in hepatocellular carcinoma diagnosis without prior EV isolation (98). Other one-pot approaches, including rolling circle amplification in encoded hydrogel microparticles and direct RT-qPCR-based EV-miRNA assays, further reduce sample handling and avoid losses associated with standard RNA purification (98). Microfluidic and sensor-integrated platforms provide another major route toward streamlined EV analysis. These “lab-on-a-chip” systems can combine EV enrichment, washing, lysis, and signal readout within a single device, reducing sample volume requirements and improving reproducibility. Immunoaffinity-based microfluidic chips, such as MITEV, have been used to enrich tumor-derived EVs from pancreatic cancer plasma and enable downstream mutational analysis, including KRAS detection. Size-based microfluidic platforms such as ExoFAST offer automated enrichment of small EVs with performance comparable to conventional ultracentrifugation-based workflows (99). In parallel, integration with surface-enhanced Raman spectroscopy, electrochemical sensors, or plasmonic biosensors has enabled highly sensitive EV protein or molecular profiling in small-volume clinical samples. For example, SERS-based microfluidic systems have been applied to direct small-EV profiling in cerebrospinal fluid, supporting the diagnosis and monitoring of pediatric brain tumors (100, 101).

Despite these advances, separation-free and integrated EV assays still face several translational barriers. These include matrix interference from complex biofluids, variability in antibody or aptamer capture efficiency, difficulty distinguishing tumor-derived EVs from abundant background EVs, and limited cross-platform standardization. Future development should prioritize analytical validation, standardized reporting, automation, and prospective testing in clinically relevant cohorts.

### 5.2 Spatial liquid biopsy and near-source EV sampling

Spatial liquid biopsy and near-source sampling strategies focus on analyzing EVs from anatomical sites or biofluids that are in closer proximity to the disease locus, rather than relying solely on systemic circulation like peripheral blood. This approach capitalizes on the premise that “near-source” fluids contain a higher concentration of disease-specific EVs with a stronger tumor or pathological signal, potentially offering greater diagnostic sensitivity and specificity for early detection and localized disease monitoring. In the field of gastroenterology and hepatopancreatobiliary diseases, endoscopy-guided liquid biopsy has emerged as a powerful tool. Procedures such as endoscopic retrograde cholangiopancreatography (ERCP) or endoscopic ultrasound (EUS) allow for the direct collection of pancreatic juice, bile, duodenal fluid, or even portal venous blood. These fluids are in direct contact with or drain from organs like the pancreas, where EVs shed by early neoplastic lesions can be concentrated before significant dilution into the systemic circulation. For PDAC, which is notoriously difficult to diagnose early, analyzing EVs from pancreatic juice or bile presents a unique opportunity. Studies have explored the potential of EV-carried molecules, including proteins and miRNAs, as early detection biomarkers for pancreatic cancer, although none have yet been validated for clinical use (102). Machine learning pipelines integrating EV characteristics with clinical data from such near-source samples have shown high accuracy in stratifying PDAC patient risk, highlighting the diagnostic value of this spatial approach (103). Similarly, for hepatocellular carcinoma (HCC), bile-derived EVs may provide a richer source of tumor-specific markers compared to peripheral blood, complementing the detection of circulating EV proteins like Glypican-3. The minimally invasive nature of endoscopic procedures, compared to surgical biopsy, makes repeated sampling feasible for monitoring disease progression or treatment response, aligning with the principles of precision oncology.

For diseases of the central nervous system (CNS) and other localized pathologies, analysis of CSF and other local body fluids provides a direct window into the pathological microenvironment. CSF, which bathes the brain and spinal cord, is a more proximal and enriched source of CNS-derived EVs compared to blood, where their concentration is significantly lower due to the blood-brain barrier. This makes CSF an ideal biofluid for diagnosing and monitoring brain tumors and neurodegenerative diseases. For instance, direct SERS profiling of small EVs in CSF has enabled highly accurate detection and discrimination of pediatric brain tumors like medulloblastoma, and allowed for molecular-level monitoring of therapeutic response (104). Another study developed a multiplexed RNA profiling platform for direct analysis of circulating RNAs in EVs from blood plasma, which successfully established composite signatures for accurate blood-based glioblastoma diagnosis and subtyping, though CSF remains a more direct source (105). The concept of the “neurosecretome”-encompassing all brain-derived circulating particles-highlights the potential of CSF EVPs (extracellular vesicles and particles) for liquid biopsy applications in neurological conditions, including addiction, depression, and neurodegenerative diseases like Alzheimer’s (106). CNS-derived EVs in CSF carry key biomolecules involved in Alzheimer’s pathology, such as amyloid-β and tau proteins, positioning them as promising minimally invasive biomarkers for early detection (107). Beyond the CNS, other localized effusions or lavage fluids offer similar advantages. For example, pleural effusions in lung cancer, peritoneal ascites in ovarian cancer, and bronchoalveolar lavage fluid (BALF) in pulmonary diseases contain EVs that reflect the local tumor microenvironment or inflammatory state more accurately than blood. Analysis of EV-miRNAs directly from mouse BALF and serum using a single-step RT-qPCR assay demonstrates the practicality of using such local fluids for research and potential diagnostics. Similarly, urinary EVs, primarily released from kidney cells, provide a non-invasive source for diagnosing kidney diseases, as they mirror the pathophysiological state of the renal tract (108). These spatial liquid biopsy approaches, by leveraging proximal biofluids, mitigate the dilution effect and background noise inherent in systemic blood analysis, thereby enhancing the signal-to-noise ratio for detecting disease-specific EV signatures and enabling more precise, localized pathological insights.

## 6 Future prospects and the application outlook of multi-omics integration

### 6.1 From single biomarkers to multiparametric integrated diagnostic models

The future trajectory of EVs as liquid biopsy biomarkers is unequivocally shifting from the analysis of single analytes towards the construction of integrated, multiparametric diagnostic and prognostic models. This evolution is driven by the inherent heterogeneity of EVs, which carry a diverse molecular cargo-including proteins, various RNA species, DNA, and lipids-that collectively offers a more comprehensive snapshot of the originating cell’s physiological or pathological state (109). By leveraging multiple types of molecules, these integrated models can overcome the limitations posed by tumor heterogeneity, providing a more robust and holistic reflection of disease (110). For instance, studies are increasingly utilizing machine learning to fuse EV-derived data with conventional clinical parameters. A prime example is in HCC, where a diagnostic model integrating an EV lncRNA score (derived from KCNQ1-AS1 and LINC01785) with clinical variables like age and alpha-fetoprotein (AFP) levels achieved an AUC exceeding 0.90, significantly outperforming AFP alone (111). Similarly, in prostate cancer (PCa), a urine EV-based 3-lncRNA classifier demonstrated superior accuracy for detecting high-grade disease compared to established tools like PCA3 or multiparametric MRI, and it also served as an independent predictor for disease progression during active surveillance (112). The power of multi-omics integration is further highlighted in research identifying collagen-related signatures; machine learning models built from transcriptomic, proteomic, and urinome (EV) data related to collagen metabolism showed comparable performance to MRI in detecting clinically significant PCa, successfully identifying cases even among equivocal imaging lesions (113). This approach of cotargeting multiple biomarkers, such as different tetraspanins on EV surfaces, is recognized as a strategy to enhance detection sensitivity by accounting for EV heterogeneity (114). Computational frameworks and artificial intelligence are now pivotal in systematically selecting and validating these multiparametric EV signatures from vast omics datasets, ensuring biological plausibility and clinical utility to accelerate translation (115). Ultimately, the transition to multiparametric models signifies a move towards more accurate, reliable, and finely stratified patient management, harnessing the full informational potential of EVs to mirror the complex biology of disease.

### 6.2 Deepening applications across the precision oncology workflow

The application of EV-based liquid biopsies is poised to deepen across the entire continuum of cancer care, from initial screening to therapy monitoring and understanding treatment resistance. For early screening and diagnosis, the development of ultra-sensitive detection platforms is critical. Advanced technologies like Bessel beam excitation separation and multiparametric biochip assays enable high-throughput, single-EV analysis, facilitating the discovery of biomarkers in accessible biofluids such as saliva and plasma for cancers like gastric cancer (116). Microfluidic platforms, such as the 3D DynaMag-EV capture chip, allow for rapid and efficient EV enrichment from plasma, enabling the identification of early-stage HCC-specific lncRNAs even in AFP-negative cases. The exploration of proximal fluids like urine, ascites, and utero-tubal lavage fluid also offers rich sources of EV biomarkers for cancers such as ovarian cancer, potentially enabling detection before imaging reveals a lesion (117). For real-time dynamic monitoring, EVs offer a minimally invasive means to track disease burden and treatment response. In prostate cancer radiotherapy, EVs are investigated as biomarkers to monitor treatment efficacy and early signs of recurrence, providing a dynamic tool for adjusting therapeutic strategies (118). The analysis of EV cargo, such as specific proteins or miRNAs, can reflect the tumor’s response to therapies, including immunotherapy, by offering insights into the evolving tumor microenvironment (119). This capability for serial assessment is a cornerstone of liquid biopsy, allowing for comprehensive quantification of whole-body tumor burden over time (120). Regarding research into resistance mechanisms and overcoming them, EVs are instrumental in non-invasively exploring new pathways of therapy resistance. By analyzing changes in EV cargo before and after treatment, researchers can identify molecular signatures associated with radioresistance or chemoresistance. For example, in cancers treated with immune checkpoint inhibitors, tumor-derived EVs carry neoantigens and molecular signatures that reflect the tumor’s genetic complexity and immune interactions, providing clues to mechanisms of immune evasion and treatment failure. This knowledge can then guide the selection of subsequent therapeutic strategies. The utility of EVs extends across various pediatric and adult solid tumors, including neuroblastoma, sarcomas, and renal cell carcinoma, where they are studied for diagnosis, minimal residual disease monitoring, and prognosis prediction (121–123). Thus, the integration of EV analysis promises to refine every step of oncology practice, enabling earlier intervention, personalized treatment adaptation, and insights into therapeutic challenges.

### 6.3 Beyond oncology: potential in chronic diseases and health management

**Figure 3:**
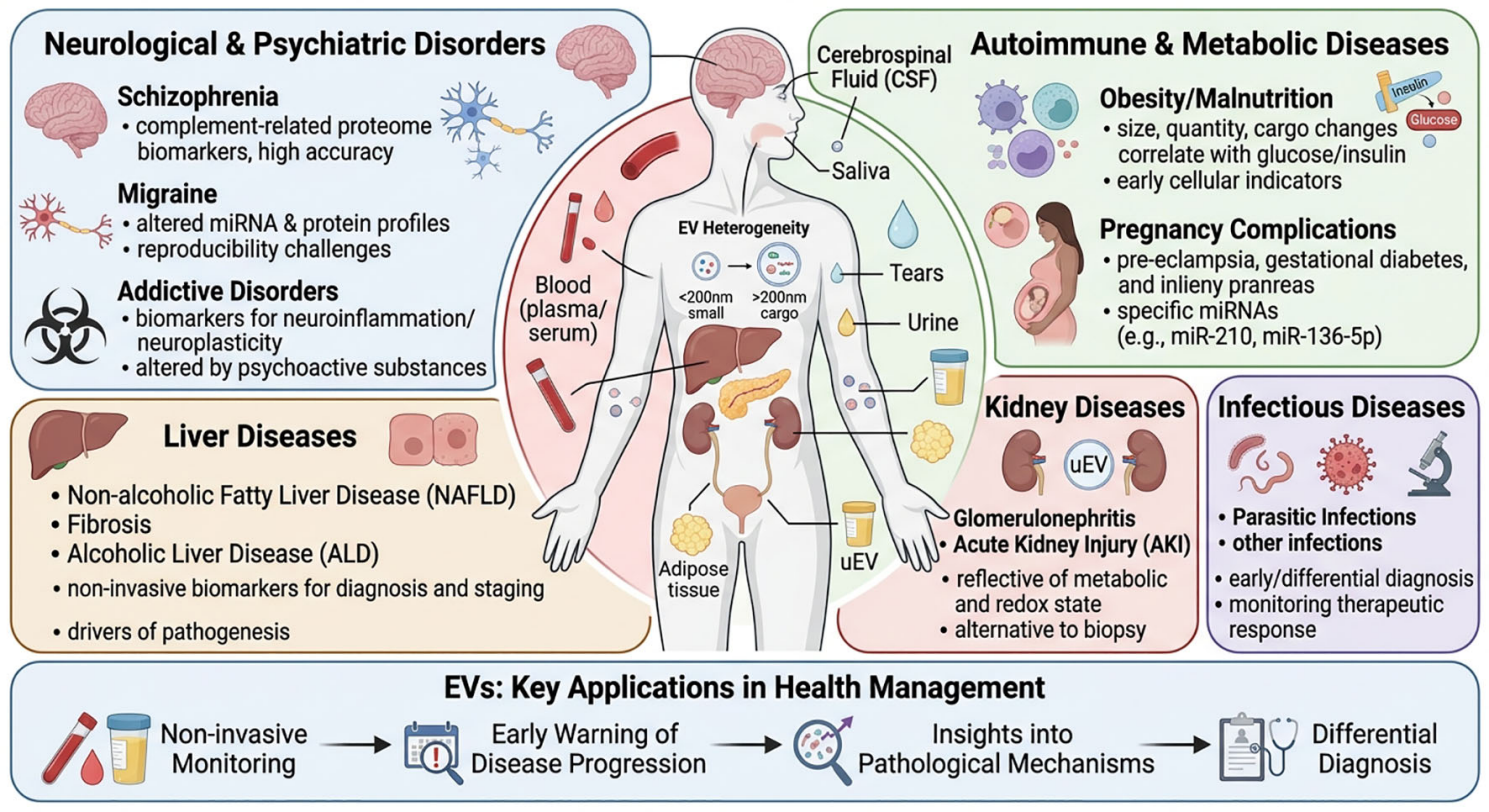
Potential applications of EVs in non-oncological conditions. EVs are versatile liquid biopsy biomarkers beyond oncology, offering a non-invasive window into chronic diseases and health management. Derived from diverse organs and detectable in multiple biofluids, EVs carry RNAs, proteins, and lipids that reflect pathological changes in neurological, liver, metabolic, kidney, and infectious diseases, supporting early diagnosis, disease monitoring, and mechanistic insight.

The utility of EV-based liquid biopsy is rapidly expanding beyond the realm of oncology, demonstrating significant potential for the management of chronic diseases and general health monitoring (**Figure 3**). In neurodegenerative and neuropsychiatric disorders, EVs derived from biofluids like blood and cerebrospinal fluid offer a window into central nervous system pathology. For schizophrenia, EV proteome profiling has identified complement-related biomarkers that outperform plasma-based markers, achieving high accuracy in distinguishing patients from healthy controls and differentiating schizophrenia from other disorders like bipolar depression (124). EVs are also investigated as potential biomarkers and therapeutic carriers in migraine, where alterations in EV miRNA and protein profiles may reflect pathological states, though reproducibility remains a challenge (125). Their role in addictive disorders is being explored, as psychoactive substances alter EV biogenesis and cargo, suggesting EVs as potential biomarkers for neuroinflammation and neuroplasticity changes associated with addiction (126). (157) For autoimmune and metabolic diseases, EVs carry disease-associated proteins that could serve as non-invasive screening tools (127). In conditions of malnutrition, such as obesity, changes in the size, quantity, and content of EVs correlate with metabolic parameters like glucose and insulin, indicating their involvement in the development of metabolic complications and their potential as early indicators of cellular alterations (128). EVs are also implicated in pregnancy complications like pre-eclampsia and gestational diabetes, with specific EV-carried miRNAs (e.g., miR-210, miR-136-5p) identified as potential biomarkers, though clinical application requires further validation (129). In the context of organ injury and chronic diseases, EVs show great promise. In liver diseases, including non-alcoholic fatty liver disease, fibrosis, and alcoholic liver disease, hepatocyte-derived EVs are studied as drivers of disease pathogenesis and as non-invasive biomarkers for diagnosis and staging, addressing the current reliance on invasive biopsy (130, 131). For kidney diseases, uEVs are a prime source of biomarkers for conditions like glomerulonephritis and acute kidney injury, reflecting the metabolic and redox state of kidney cells and offering an alternative to invasive kidney biopsy (132, 133). Furthermore, EVs are emerging as diagnostic tools in infectious diseases, including parasitic infections, where EV cargo analysis can identify biomarkers for early and differential diagnosis, and in monitoring therapeutic responses (134). The exploration of diverse biofluids, including tears and saliva, further broadens the scope of EV-based liquid biopsy for systemic and organ-specific conditions (135). This expansion into chronic disease management underscores the versatility of EVs as biomarkers, enabling non-invasive monitoring, early warning of disease progression, and insights into underlying pathological mechanisms across a wide spectrum of human health.

## 7 Conclusion

EVs are emerging as powerful liquid biopsy biomarkers because of their stable lipid bilayer, rich multi-omic cargo, and ability to reflect the molecular state of parent cells and disease microenvironments. They hold substantial promise for early diagnosis, molecular subtyping, treatment response monitoring, recurrence prediction, and personalized disease management, particularly in cancer. However, clinical translation remains limited by EV heterogeneity, non-standardized isolation and analytical workflows, insufficient reference materials, and limited large-scale prospective validation. Future progress requires a balanced strategy that combines methodological standardization with continued technological innovation. International guidelines, standardized protocols, and benchmarked reference materials are essential to improve reproducibility and enable cross-study comparison. Meanwhile, emerging approaches such as separation-free detection, single-vesicle analysis, microfluidics, near-source sampling, and integrated biosensors may improve sensitivity, speed, and clinical feasibility. Importantly, EV-based diagnostics should move beyond single biomarkers toward integrated multi-omics profiling of proteins, nucleic acids, lipids, and metabolites. Coupled with artificial intelligence and machine learning, such multidimensional EV signatures may unlock their full potential as clinically actionable tools in precision medicine.
